# Association between health literacy and self-care behaviors among patients with chronic kidney disease

**DOI:** 10.1186/s12882-018-0988-0

**Published:** 2018-08-06

**Authors:** Karen K. Wong, Alexandra Velasquez, Neil R. Powe, Delphine S. Tuot

**Affiliations:** 1 Touro University California College of Osteopathic Medicine, Vallejo, USA; 2 Division of Nephrology, University of California, San Francisco, USA; 3 Department of Medicine, University of California, San Francisco, USA; 4 Center for Vulnerable Populations, University of California, San Francisco, USA; 5 Priscilla Chan and Mark Zuckerberg San Francisco General Hospital and Trauma Center, University of California, 1001 Potrero Ave, Bldg. 100, Room 342, San Francisco, CA 94110 USA

**Keywords:** Health literacy, Chronic kidney disease, Self-management support, Self-care, CKD

## Abstract

**Background:**

We explored the association between health literacy and self-care behaviors among low-income patients with chronic kidney disease (CKD).

**Methods:**

We used baseline data from the Kidney Awareness Registry and Education trial (*n* = 137 patients with CKD) and multivariable logistic regressions to cross-sectionally examine the association between health literacy, defined by a validated questionnaire, and healthy behaviors.

**Results:**

Study participants had a mean age of 55 years, were racially diverse (6% White, 36% Hispanic, 43% Black, 15% Asian) and 26% had low health literacy. Over one-third (38%) had hypertension, 51% had diabetes, and 67% had CKD stage 3 or 4. Compared to individuals with adequate health literacy, those with low health literacy had non-statistically significant higher tobacco use (adjusted odds ratio [aOR] = 2.33; 95% CI 0.90–6.06) and lower consumption of sugary beverages (aOR = 0.50; 0.20-1.23) and statistically significant decreased fast food intake (aOR = 0.38; 0.16-0.93). Health literacy was not associated with differences in medication adherence (0.84; 0.38-1.89) or physical activity (aOR = 2.39; 0.54-10.53).

**Conclusions:**

Health literacy was not uniformly associated with all self-care behaviors important for CKD management. A more nuanced understanding of the association of health literacy and self-care may be necessary to promote participation in behaviors known to slow CKD progression.

## Background

Chronic kidney disease (CKD) is a prevalent health condition that affects nearly 15% of the U.S population [1]. CKD is associated with higher risks of cardiovascular disease (CVD), premature mortality, and decreased quality of life [2]. Self-care behaviors are generally associated with improved health among individuals with chronic diseases. Physical activity has been associated with improved cardiovascular outcomes among patients with diabetes and CKD; smoking cessation, decreased alcohol consumption, and maintaining a healthy body mass index have been shown to significantly reduce incidence of proteinuria [3–5]. Participation in self-care behaviors may thus represent one way to mitigate adverse outcomes associated with CKD.

Engaging in self-care activity is commonly regarded as the proximal outcome of awareness/understanding of chronic health conditions [6]. It is well established that individual CKD awareness is low in the United States. Data from the 1999–2012 National Health and Nutrition Examination Survey (NHANES) estimated that overall awareness of CKD status among community-dwelling adults was 6.4% [7]. Similar results have been noted among cohort participants from the Kidney Early Evaluation Program (KEEP) [8] and Reasons for Geographic and Racial Differences in Stroke (REGARDS) study [9], as well as patients followed in clinical practice [10]. The association between patient awareness of kidney disease and participation in self-care activities, however, is less clear. Patients with CKD followed in a nephrology clinic aware of blood pressure (BP) goals have been shown to have improved BP compared to those unaware [11]. Patients with ESRD aware of chronic comorbid conditions have been shown to have lower mortality risk compared to those unaware [12]. On the other hand, awareness of CKD among participants in the REGARDS study was not associated with greater participation in self-care activities or improved BP [9].

Current literature suggests that health literacy may be an important factor in the care of patients with kidney diseases and may influence the impact of CKD awareness on patient participation in healthy behaviors [13, 14]. U.S. Health Resources and Services Administration defines health literacy as “the degree to which individuals have the capacity to obtain, process and understand basic health information needed to make appropriate health decisions and services needed to prevent or treat illness” [15]. Low health literacy is common among patients with CKD and end stage renal disease (ESRD) and has been associated with less knowledge about kidney function and dialysis [16]. Among patients with ESRD, higher health literacy has been associated with greater participation in self-management behaviors such as medication adherence and phosphate control [17]. Comparable studies among patients with CKD are lacking, however. Our study was designed to examine the relationship between health literacy and participation in selected self-care behaviors among patients with CKD. We hypothesized that low health literacy would be associated with decreased engagement in self-care behaviors, potentially offering an explanation as to why CKD awareness among patients may not always translate into participation in healthy behaviors.

## Methods

### Study design and participants

**Fig. 1:**
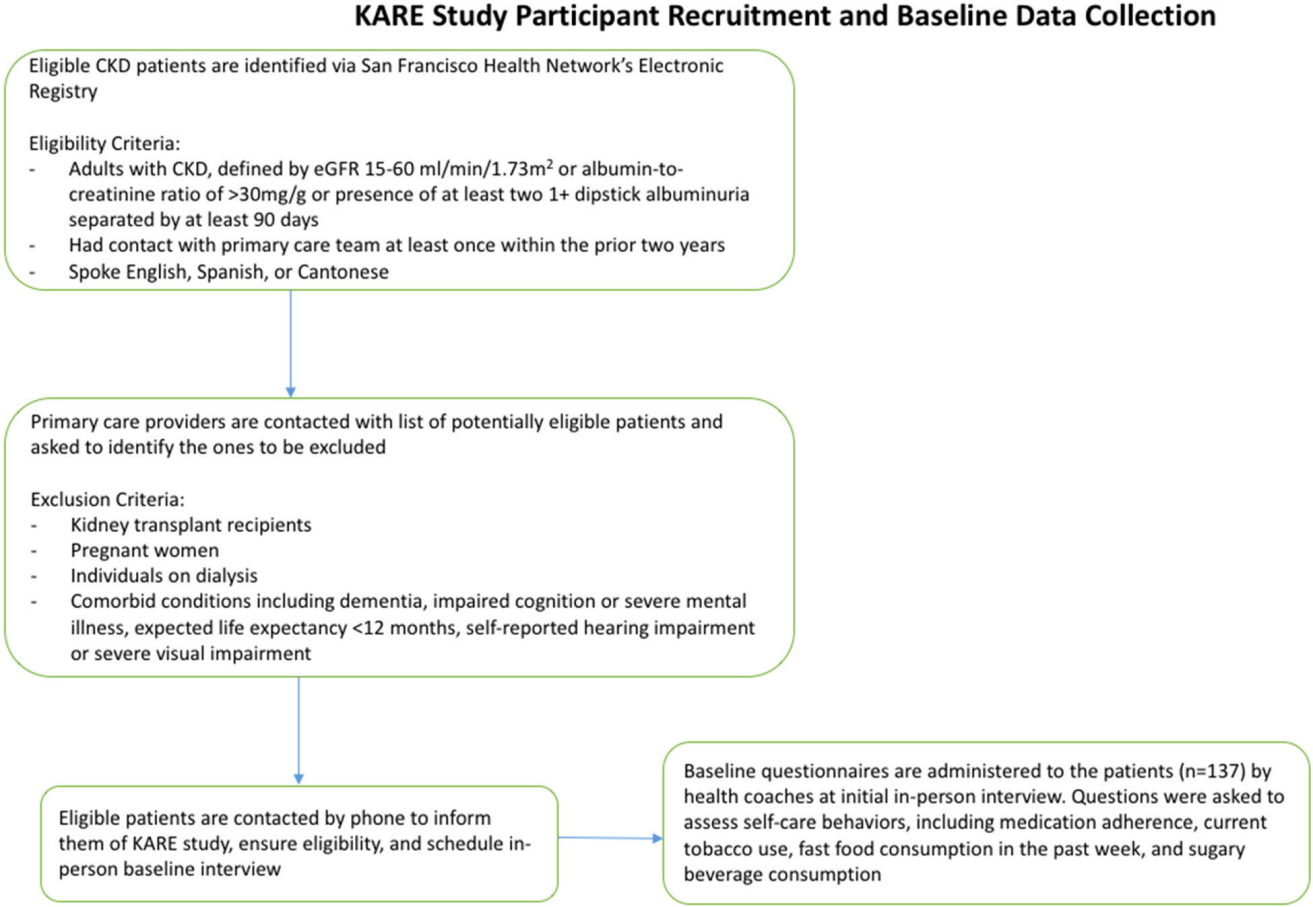
Study flow diagram

This was a retrospective cross-sectional study using baseline data from the Kidney Awareness Registry and Education (KARE) study, a pilot randomized controlled trial (NCT01530958) examining the impact of a language-concordant and culturally sensitive CKD self-management support (CKD-SMS) program on blood pressure control among diverse patients with CKD who received primary care in urban safety-net clinics [18]. As this was an ancillary study to KARE; all pre-randomization data from the 137 KARE participants related to demographics, health literacy, and self-reported participation in self-care behaviors were used. Details of KARE participant eligibility and recruitment have been previously reported. In brief, the San Francisco Health Network’s electronic patient registry was used to identify eligible patients, who included adults with CKD, defined by an estimated glomerular filtration rate (eGFR) 15–60 ml/min/1.73 m^2^ or albumin-to-creatinine ratio of > 30 mg/g or presence of at least two ≥ 1+ dipstick albuminuria separated by at least 90 days, who had contact with their primary health care team at least once within the prior 2 years and spoke English, Spanish or Cantonese. Exclusion criteria included: kidney transplant recipients, pregnant women, individuals on dialysis, and presence of co-morbid conditions that impeded meaningful communication between providers and patients or limited the usefulness of a self-management support program: prevalent dementia, impaired cognition or severe mental illness; expected life expectancy < 12 months; self-reported hearing impairment or severe visual impairment preventing use of a touchtone telephone keypad (Fig. 1). After the baseline visit, KARE participants were randomized to one of two groups: usual care without intervention or participation in a comprehensive self-management support program in addition to usual care. The study was approved by the UCSF Institutional Review Board (Protocol # 11–07399).

### Measurements

Baseline demographic data were self-reported and included: age, gender, race, preferred language, education attainment, and health insurance. Comorbid conditions (hypertension, diabetes, mean eGFR, and proteinuria) were ascertained from the electronic health record. Health literacy and self-care behaviors (medication adherence, tobacco use, activity level, fast food consumption, and sugary beverage intake) were ascertained with validated questionnaires [19–21].

Health literacy, the primary explanatory variable, was determined by a validated 3-item screening questionnaire that assesses the patient’s ability to comprehend health information. Questions include: 1. “How often do you have someone help you read hospital materials?” 2. “How confident are you filling out medical forms by yourself?” 3. “How often do you have problems learning about your medical condition because of difficulty understanding written information?” Patients indicated their responses to each question on a 5-point scale from 0 to 4. The score for each item was summed and the total divided by 3. An individual was considered to have limited health literacy if he/she had a score ≥ 6 out of a maximal score of 12 [19].

Outcomes included measures of selected self-care behaviors: medication adherence, tobacco use, physical activity, fast food consumption, and sugary beverage intake. Medication adherence was assessed with a 4-item validated, subjective scale of medication adherence [20]: 1. “Do you ever forget to take your medicine?” 2. “Are you careless at times about taking your medicine?” 3. “When you feel better, do you sometimes stop taking your medicine?” 4. “Sometimes if you feel worse when you take the medicine, do you stop taking it?” Responses were either Yes or No to each question, which a score of 1 for each “Yes”. Responses were categorized as “high adherence” – 0 points; “intermediate adherence” – 1-2 points; or “low adherence” – 3-4 points. Current tobacco use was determined using 2 validated questions from the 2009 Behavioral Risk Factor Surveillance Survey [21]: 1. “Have you smoked at least 100 cigarettes in your entire life? (5 packs=100 cigarettes)” (response options were “yes”, “no”, and “don’t know/not sure”) and 2. “Do you now smoke cigarettes every day, some days, or not at all?” (response options were “every day”, “some days”, “not at all”, and “don’t know/not sure”). Responses to both questions were used to create a binary variable depicting current tobacco use. Participation in physical activity was also determined using three items from the Behavioral Risk Factor Surveillance System (BRFSS): (1) “When you are at work, which of the following best describes what you do?” Answer options were categorized as none/light activity [mostly sitting or standing or mostly walking] or moderate/vigorous activity [mostly heavy labor or physically demanding work]; (2) Thinking about the moderate activities you do when you are not working in a usual week, do you do moderate activities for at least 10 min at a time, such as brisk walking, bicycling, vacuuming, gardening, or anything else that causes some increase in breathing or heart rate? Answers options were “yes”, “no”, or “don’t know/not sure”; and (3) Thinking about the vigorous activities you do when you are not working in a usual week, do you do vigorous activities for at least 10 min at a time, such as running, aerobics, heavy yard work, or anything else that causes large increases in breathing or heart rate? Answer options were “yes”, “no”, or “don’t know/not sure” [21]. Fast food and sugary beverage consumption were also assessed using questions from BRFSS. Individuals were asked to indicate the number of times in the past week they had eaten from a fast food restaurant (“During the past 7 days, how many meals did you eat from a fast-food restaurant or pizza place?”) and the number of times per day/week/month/year that they consumed sugary beverages (“How often do you drink soda that contains sugar? Do not include diet soda” and “How often do you drink sweetened fruit drinks, such as Kool-Aid and lemonade? Include fruit drinks you make at home and add sugar to.” Responses for fast food intake were categorized as “none”, “once”, “twice” or “three or more”. Responses for sugary beverage intake were categorized into: “never”, “monthly/yearly” or “daily/weekly” [21].

### Statistical analysis

Participant characteristics were compared by level of health literacy by chi-square and ANOVA tests. Multivariable logistic regression models were used to determine odds ratios and 95% confidence interval for the presence, direction, strength and independence of an association of literacy level with current tobacco use and low physical activity. Ordinal logistic regression models were used to determine the association of health literacy status with lower medication adherence, greater fast food intake and higher sugary beverage consumption. Each model was adjusted for confounders statistically associated with both the explanatory variable and the outcomes in this study: age, diabetes, and educational attainment, as well as race and gender to be consistent with prior literature. Sample size calculations suggested that with estimated prevalence of limited health literacy and tobacco use both around 40% for this study population (unpublished data), a sample size of 137 participants would provide 80% power to detect a 2.8-fold increased odds of tobacco use among those with limited health literacy compared to those with adequate health literacy. All statistical analyses were performed using STATA software, Version 14.0 (StataCorp, College Station, Texas, USA).

## Results

### Participant characteristics

Among the 137 study participants, 101 (74%) had adequate health literacy and 36 (26%) had low health literacy. Individuals with low health literacy were older than those with adequate health literacy (mean age 59.4 years vs. 53.9 years, *p* = 0.02), but were similar with respect to gender, race/ethnicity, language preference and health insurance. The overall study population was racially diverse (6% White, 36% Hispanic, 43% Black, and 15% Asian) and nearly 40% had a non-English language preference. Nearly 25% had no health insurance, 45% had Medi-Caid, and 27% had Medi-Care. Individuals with low health literacy had less educational attainment than those with adequate health literacy, with higher proportions of individuals reporting only completing primary school and fewer reporting attending college (*p* = 0.003). A greater proportion of individuals with low literacy had diabetes compared to those with adequate health literacy (75% vs. 44%, *p* < 0.001), though the two groups of participants did not differ with respect to the prevalence of hypertension, prevalence of albuminuria or severity of CKD. Overall, 39% of the study population had hypertension, 69% had albuminuria 33% had Stage 1 or 2 CKD, and 67% had Stage 3 or 4 CKD (Table 1).

**Table 1:** Characteristics of study participants

Characteristics	All	Low health literacy	Adequate health literacy	*p*-value
	N = 137	*N* = 36	*N* = 101	
Age, mean (SD)	55.32 (12.2)	59.44 (10.4)	53.86 (12.6)	0.02
Male, % (N)	48.18 (66)	41.67 (15)	50.50 (51)	0.36
Race/ethnicity, % (N)				0.87
Non-Hispanic White	5.88 (8)	5.56 (2)	6.00 (6)	
Hispanic	36.03 (49)	30.56 (11)	38 (38)	
Black	43.38 (59)	47.22 (17)	42 (42)	
Asian	14.71 (20)	16.67 (6)	14 (14)	
Language preference, % (N)				0.29
English	61.31 (84)	58.33 (21)	62.38 (63)	
Spanish	32.85 (45)	30.56 (11)	33.66 (34)	
Cantonese	5.84 (8)	11.11 (4)	3.96 (4)	
Educational attainment, % (N)				0.003
Primary school	13.87 (19)	27.78 (10)	8.91 (9)	
High school or technical school	51.82 (71)	55.56 (20)	50.50 (51)	
College	34.31 (47)	16.67 (6)	40.59 (41)	
Health insurance^a^, % (N)				0.25
None	24.82 (34)	16.67 (6)	27.72 (28)	
Medi-Caid	44.53 (61)	50.00 (18)	42.57 (43)	
Medi-Care	27.01 (37)	33.33 (12)	24.75 (25)	
Co-morbid conditions				
Hypertension, % (N)	38.69 (53)	33.33 (12)	40.59 (41)	0.44
Diabetes, % (N)	51.82 (71)	75.00 (27)	43.56 (44)	< 0.001
eGFR, mean (SD)	46.28 (9.7)	46.85 (7.75)	46.1 (10.43)	0.73
CKD Stages, % (N)				0.45
Stage 1/2	32.85 (45)	27.78 (10)	34.65 (35)	
Stage 3/4	67.15 (92)	72.22 (26)	65.35 (66)	
Albuminuria > 30 mg/g, % (N)	69.34 (95)	72.22 (26)	68.32 (69)	0.66

^a^Other category not shown

### Prevalence of self-care behaviors

Over two-thirds of study participants (70%; *n* = 95) reported lower medication adherence (51%, *n* = 70 with intermediate adherence; 18.2%, *n* = 25 with low adherence) and nearly one-third (32.6%, *n* = 44) reported current tobacco use, neither of which differed by health literacy level. Relatively few study participants reported engaging in no or light activity (11.8%, *n* = 16), again with similar levels of engagement by health literacy status (*p* = 0.50). Overall, a small number of study participants (14.6%) reported frequent fast food consumption, though healthier patterns were patient-reported among those with limited health literacy (*p* = 0.04). Similarly, while 60% of all study participants reported daily or weekly sugary beverage consumption, healthier patterns were reported by individuals with low health literacy (*p* = 0.01) (Table 2).

**Table 2:** Prevalence of self-care behaviors among study participants

Self-care behaviors	All	Low health literacy	adequate health literacy	*p*-value
	*N* = 137	*n* = 36	*n* = 101	
Medication adherence, % (n)				0.71
Low	18.3 (25)	16.7 (6)	18.8 (19)	
Intermediate	51.1 (70)	47.2 (17)	52.5 (53)	
High	30.7 (42)	36.1 (13)	28.7 (29)	
Current tobacco use. % (n)	32.6 (44)	42.9 (15)	29.0 (29)	0.13
No activity or light activity level, % (n)	11.8 (16)	8.6 (3)	12.9 (13)	0.50
Fast food consumption in past week, % (n)				0.041
None	38.69 (53)	58.33 (21)	31.68 (32)	
Once	36.50 (50)	27.78 (10)	39.60 (40)	
Twice	10.22 (14)	5.56 (2)	11.88 (12)	
Three or greater	14.60 (20)	8.33 (3)	16.83 (17)	
Sugary beverage consumption, % (n)				0.01
Never	22.96 (31)	40.00 (14)	17.00 (17)	
Monthly/Yearly	18.52 (25)	22.86 (8)	17.00 (17)	
Daily/Weekly	58.52 (79)	37.14 (13)	66.00 (66)	

### Self-care behaviors and health literacy

**Fig. 2:**
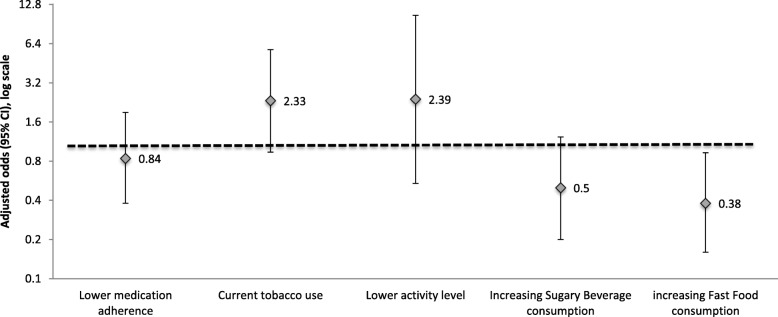
Odds of participation in self-care behaviors among individuals with low (vs adequate) health literacy status, adjusted for age, sex, race/ethnicity, educational attainment and diabetes status

Among KARE study participants, health literacy was not associated with lower medication adherence (adjusted odds ratio [aOR] = 0.84; 95% CI 0.38–1.89) or physical activity (aOR = 2.39; 0.54–10.53), independent of age, gender, race/ethnicity, educational attainment and diabetes status (Fig. 2). However, patients with lower health literacy had non-statistically significant higher tobacco use compared to those with adequate health literacy (aOR = 2.33; 0.90–6.06). Additionally, individuals with low health literacy reported substantially lower consumption of sugary beverages (aOR = 0.50; 0.20–1.23) and statistically significant decreased fast food intake (aOR = 0.38; 0.16–0.93) compared to those with high health literacy.

## Discussion

Our data confirm that limited health literacy is common among low-income patient populations with CKD [22] and suggest that adequate health literacy is not associated with greater engagement in all self-care behaviors that are critical to the management of CKD. More specifically, we demonstrate that in a low income, safety-net population with CKD, low health literacy is associated with higher tobacco use (though not statistically significant) but statistically significant better dietary habits, and that it is not associated with medication adherence or physical activity. In order to unify these results, one must consider that health literacy may be important but insufficient to lead to participation in healthy behaviors and that other factors are likely to be more influential in CKD self-management.

One such factor is knowledge or perception of how a behavior directly impacts kidney disease. Prior studies have demonstrated that patients with less knowledge of smoking health risks perceive themselves as less vulnerable to health consequences of smoking and demonstrate less intent to change their smoking behavior [23, 24]. Similarly, a study among diabetic patients noted that participation in education classes that focused on the association between diet and diabetes led to better adherence to healthier diets among those with low health literacy, despite there not being any association prior to the educational endeavor [25]. We did not ascertain prior receipt of education about tobacco or healthy diets, but it’s plausible that participating patients with lower health literacy perceived healthier diets as more directly relevant to kidney and overall health than tobacco use from prior educational discussions with members of the primary care team. Diet is now recognized as one of the important factors controlling chronic disease progression, and health providers often focus on dietary behavior change while providing patient education. It’s plausible that this was more enforced among patients with low health literacy. Prior to the KARE study, there had not been any formal educational interventions for patients with kidney disease in the health delivery system in which this study took place. There were, however, many opportunities for patients to learn about healthy self-management behaviors, including appointments with a primary care nutritionist, referrals to tobacco cessation classes, and access to low-literacy written educational materials available to providers in the electronic health record to hand out to patients [26]. It is possible that patients with lower health literacy were offered more chances to participate in these opportunities, which may have positively impacted their dietary habits.

Trust in physicians and the healthcare system is another factor that may influence the association between health literacy and participation in self-care behaviors, particularly medication adherence. A recent meta-analysis examining the relationship between health literacy and medication adherence among 35 studies found a small but significant positive relationship, with a 14% increased risk of non-adherence among individuals with low health literacy [27]. This makes intuitive sense, as low health literacy among primary care patients has been identified as an independent predictor of incorrect interpretation of prescription warning labels, contributing to non-adherence and therapeutic failure [28]. However, in a study examining self-care behaviors among low-income adults with diabetes, low literacy was associated with greater (though non-statistically significant) adherence to medication regimens, thought to be mediated through greater trust in physicians [29]. Similarly, medication adherence among elderly patients in another study was associated with greater patient satisfaction with medication counseling and receipt of medication explanations, rather than health literacy [30]. We did not specifically ascertain trust in the healthcare system in our study, but it is plausible that participants had greater trust in their provider than the average primary care patient since they enrolled in a clinical trial. Our findings suggest that health literacy may be just one contributor to medication adherence and that other critical factors that impact medication adherence among patients with CKD, likely exist.

It is also possible that numeracy may play a more direct role in guiding participation in self-management behaviors than health literacy. Numeracy is broadly defined as the ability to use numbers in daily life. While numeracy is an important component of literacy in general, it is a separate construct from health literacy in that it involves understanding calculations, dates, tables, graphs, and other skills [31]. Prior research supports that numeracy influences how individuals interpret medical risk information [32], and that health literacy and numeracy skills do not always track together in the same patients [33]. Numeracy skills are of particular importance in helping patients understand nutrition labels and may either increase or decrease the likelihood of action through information seeking, computation, and interpretation of meaning [34]. Prior studies have shown that individuals with lower numeracy skills may consume a greater caloric intake from carbohydrates and inaccurately overestimate single-serving portion sizes [35, 36]. While we did not assess numeracy in this study, it is reasonable to consider that study participants with low health literacy may have had better numeracy skills, guiding them to make healthier decisions about purchasing and consuming food and beverages.

Understanding the relationships between health literacy, health awareness/understanding, and self-care behaviors are key to better executing intervention strategies to improve health outcomes, particularly in socially challenged environments. As suggested in this study and others, these relationships are not always consistent and self-care behaviors may be more critically impacted by disease knowledge, patient-provider relationships, numeracy skills, and personal choice, than by health literacy. If this is indeed the case, then all patients with CKD regardless of literacy status would benefit from targeted health education about self-management behaviors from trusted sources in the health system. To ensure that individuals with limited health literacy benefit from such educational interventions, it would be important to include the use of simple language communication, teach-back methods, and non-written educational modalities such as video to enhance understanding and engagement in specific health behaviors.

This study has several limitations. The small sample size limits power and ability to generalize findings to other populations with CKD. Additionally, the cross-sectional nature of this study precludes conclusions about causality, just association between health literacy and self-care behaviors. All patients in this study had agreed to participate in a self-management trial, which might suggest that their willingness to perform self-care behaviors is different than the average individual with CKD. Adequacy of health literacy was defined by a validated health literacy screening tool rather than the Test of Functional Health Literacy in Adults or Rapid Estimate of Adult Literacy in Medicine. While the screening instrument has been positively correlated with these more in-depth tools, it’s possible that health literacy was under-estimated or that individuals were misclassified in their health literacy skills, thus impacting study results.

## Conclusions

In conclusion, health literacy is not uniformly associated with participation in different self-care behaviors that are critical to the management of CKD. While health literacy is regarded as a strong predictor for long-term health, it is critical to consider the underlying factors that mediate the relationship between literacy and participation in self-care activities. Such information will guide future research and provide insights in how health care teams can best intervene to promote patient participation in behaviors that slow progression of CKD and improve quality of life.

## Abbreviations

aOR: adjusted Odds Ratio
CKD: Chronic Kidney Disease
CKD-SMS: Chronic Kidney Disease Self-Management Support
eGFR: Estimated Glomerular Filtration Rate
ESRD: End Stage Renal Disease
KARE: Kidney Awareness Registry and Education
KEEP: Kidney Early Evaluation Program
NHANES: National Health and Nutrition Examination Survey
REGARDS: Reasons for Geographic And Racial Differences in Stroke
